# Germline development in amniotes: A paradigm shift in primordial germ cell specification

**DOI:** 10.1002/bies.201600025

**Published:** 2016-06-06

**Authors:** Federica Bertocchini, Susana M. Chuva de Sousa Lopes

**Affiliations:** ^1^ Institute of Biomedicine and Biotechnology of Cantabria (IBBTEC)‐CSIC‐University of CantabriaSantanderSpain; ^2^ Department of Anatomy and EmbryologyLeiden University Medical CenterLeidenThe Netherlands; ^3^ Department of Reproductive MedicineGhent University HospitalGhentBelgium

**Keywords:** amniotes, chicken, epigenesis, evolution, mammals, preformation, primordial germ cells

## Abstract

In the field of germline development in amniote vertebrates, primordial germ cell (PGC) specification in birds and reptiles remains controversial. Avians are believed to adopt a predetermination or maternal specification mode of PGC formation, contrary to an inductive mode employed by mammals and, supposedly, reptiles. Here, we revisit and review some key aspects of PGC development that channelled the current subdivision, and challenge the position of birds and reptiles as well as the ‘binary’ evolutionary model of PGC development in vertebrates. We propose an alternative view on PGC specification where germ plasm plays a role in laying the foundation for the formation of PGC precursors (pPGC), but not necessarily of PGCs. Moreover, inductive mechanisms may be necessary for the transition from pPGCs to PGCs. Within this framework, the implementation of data from birds and reptiles could provide new insights on the evolution of PGC specification in amniotes.

**Abbreviations**
BMP: bone morphogenetic protein
E: embryonic day
EG&K: developmental stage determined by Eyal‐Giladi and Kochav
HH: developmental stage determined by Hamburger and Hamilton
PGCs: primordial germ cells
pPGCs: precursor of primordial germ cells

## Introduction

The transmission of the genetic traits of an individual is ensured by the formation of special cells called germ cells: they will generate the gametes, which will confer totipotency to the newly formed zygote. The developmental path that leads to the formation of highly specialised germ cells is long and tortuous, and the molecular cues involved remain largely unknown. In animals, germ cells in the gonads produce mature gametes throughout the adult life of the organism: sperm or mature oocytes (or egg cells). One extraordinary feature in the germ cell lineage in amniotes is the fact that germ cell specification occurs far from their final location, the gonads, implying a necessary (and tightly regulated) migratory phase after specification. A second feature is their unique capacity to undergo meiosis, in which chromosome recombination generates genetic variation in the haploid gametes.

Primordial germ cells (PGCs) are uni‐potent cells capable of producing only either oocytes or spermatozoids 1. In the animal kingdom, PGCs can form via two different mechanisms: maternal specification (or predetermination) and induction (or epigenesis) (reviewed in Ref. 2).

Independent of the mode of PGC formation, the precursors of PGCs (pPGCs) are cells that progress to become PGCs. We define here somatic cells as cells that can no longer become pPGCs or PGCs 3. PGCs and pPGCs are distinct from somatic cells because they contain molecules that inhibit gene expression (transcriptional and translational repression) often concentrated in granules 4, 5, 6. Interestingly, these inhibitory factors are often species‐specific, which is suggestive of convergent evolution 2.

PGCs cannot give rise to somatic cells. By contrast, if pPGCs are transplanted to a different environment in the embryo, they can still adopt different somatic cellular fates 1, 7, 8, 9. In this sense, pPGCs may be the last cells of the gastrulating embryo that still share multipotent characteristics with the inner cell mass or the zygote. In the mouse, pPGCs became known as ‘blimped’ cells 1, 10. When (‘blimped’) pPGCs restrict their cellular fate to a PGC‐fate, they adopt a PGC‐specific transcriptional signature, undergo a severe loss of DNA methylation and increase their doubling time 11, 12. PGCs, in contrast to pPGCs, retain their cellular identity when they stray to ectopic locations during their migration 13, 14, 15. Ectopic PGCs usually undergo apoptosis, but sometimes they become either tumorigenic 15, 16 or, given the right microenvironment, they can mature as oocytes, at least in the mouse 13, 17, 18.

The idea of specification by maternal determinants (predetermination) has its roots in the pioneering work of August Weismann, who introduced the concept of ‘germ plasm’ as the substance carrying heredity 19. Maternal specification requires the presence of maternally generated determinants (mRNA) that constitute – together with high concentration of mitochondria (mitochondrial cloud) – proteins, endoplasmatic reticulum and microtubules an electron‐dense aggregate(s) known as ‘germ plasm’ or ‘nuage’. This material localises to a certain region of the oocyte and subsequently of the zygote, and then is physically inherited by the (pPGCs and) PGCs as the embryo undergoes rounds of cell division. Removal of the germ plasm from the oocyte (or zygote) typically results in the absence of PGC formation. The function of germ plasm is still not well understood, but it could confer germline identity by either suppressing somatic fate or promoting germline‐specific activity, such as specific metabolism requirements or post‐transcriptional modifications regulating certain classes of RNAs 6. Interestingly, the existence of maternal determinants seems to correlate with the timing of embryonic genome activation 3.

In the alternative mode of PGC specification, so‐called induction or epigenesis, direct induction of (pPGCs and) PGCs occurs by transiently secreted molecules produced by tissues adjacent to the site of PGC formation, that signal at a specific space and time to a relatively undifferentiated and uniform population of cells in the embryo, specifying them to PGCs.

**Figure 1:**
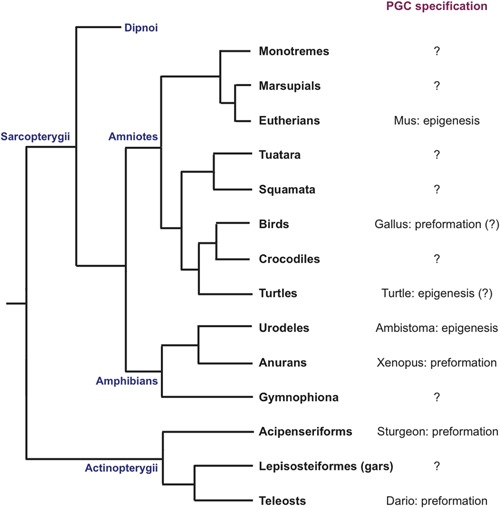
Phylogenetic tree of jawed vertebrates, with a particular emphasis on groups where PGCs have been described.

The duality between maternal specification and induction in PGC formation is a well‐accepted concept in developmental biology (Fig. 1): early embryos of some species are already mosaic, containing ‘reproductively excluded’ soma and cells that are more‐or‐less biased for germ line development, via for example germ plasm (maternal specification); whereas early embryos of other species are initially not biased, and none of the cells is ‘reproductively excluded’ (induction).

Comparative analyses between taxa lead to the current view that the ancestral mechanism of PGC formation is induction 2, 20, 21, 22, 23, with the use of maternal specification and development of germ plasm occurring in several independent events during evolution (convergent evolution). In vertebrate anamniotes, maternal specification is employed by the teleost zebrafish 24, the sturgeon 25 (which belongs to the Acipenseriforms, considered ‘primitive fish’), and amphibian anurans 26, 27. On the other hand, amphibian urodeles use induction for PGC specification 8, 28, 29, 30.

The earliest amniotes were probably derived from ancient amphibians whose features were more similar to urodeles than anurans 8, 20, 31; hence, from an evolutionary point of view, this would fit with the idea of induction being the basic mechanism of PGC specification adopted in amniotes from an urodele‐like ancestor, and diverging only in avians where supposedly maternal specification arose independently.

In amniotes, the two most representative and used experimental systems, mouse and chick, mirror this duality: mammals, and supposedly reptiles other than avians, use induction, while only birds resort to maternal specification (Fig. 1) 2. Is there enough evidence to classify amniotes according to their mode of PGC formation?

## PGC specification in mouse: The paradigm for mammals

Embryonic cell lineage specification in amniotes occurs before/around gastrulation, when the body plan coordinates are settled down and the polarities of the resulting three germ‐layered embryo (ectoderm, mesoderm, endoderm) are established. During these early events, cellular totipotency is gradually lost, and the germ line lineage is set apart from the soma.

Although the amniotes are a diversified clade, two species are used as experimental model organisms for basic laboratory research: the rodent *Mus musculus* (mouse), representing the mammals; and the avian *Gallus gallus* (chick) representing birds and reptiles (sauropsids).

Before the advent of molecular markers and transgenics, PGCs were identified by alkaline phosphatase (or Alpl) activity in mouse, and they were observed embedded in the extra‐embryonic mesoderm posterior to the mid‐primitive streak as early as embryonic day (E)7.0–7.25 32, 33. In the past 25 years, the knowledge of timing, position and, to a certain extent, the molecular machinery involved in PGC specification and further development has been refined 34, 35, 36.

**Figure 2:**
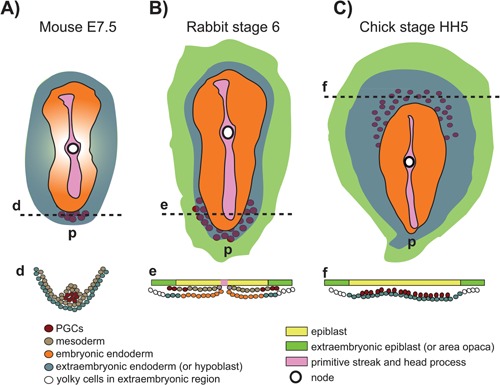
Localization of PGCs in mouse, rabbit and chick by the end of gastrulation. **A:** Mouse PGCs in the extra‐embryonic mesoderm in the posterior region (by AP staining). The mouse embryo is visualised from the distal part and flattened down for comparative purpose. **B:** Rabbit PGCs in the epiblast and in the mesoderm, (by PG‐2 epitope staining). **C:** Chick PGCs in the germinal crescent (cytological criteria and PAS colorimetric reaction). d–f, section plane depicted. p, Posterior part of the embryo.

In mouse, several Bone morphogenetic protein (Bmp) ligands, Bmp4, Bmp2 and Bmp8b were identified as inductive signals produced by adjacent extra‐embryonic regions that prime the proximal epiblast at around E6.0 to express *Ifitm3* (also known as *Fragilis*) and around E6.25, a population of about six epiblast cells (pPGCs) in the most posterior part of the proximal epiblast start expressing the transcriptional repressor *Prdm1* (or *Blimp1*). Between E6.25 and E7.25, the population of *Prdm1*‐expressing pPGCs increases from 6 to about 45, either by proliferation or induction 1, 37. *Prdm1* is not an exclusive marker of pPGCs, but is also expressed in the visceral endoderm 37. The first bona fide Alpl‐positive PGCs emerge at E7.25 in mouse (Fig. 2A) and express both pluripotency and early germline markers. In particular, the nascent PGCs are characterised by the expression of *Prdm1*, *Prdm14* and *Tfap2c* (or *Ap2γ*), transcription factors required for PGC specification at E7.25 37, 38, 39. Both *Prdm1* and *Prdm14* seem to be under the direct regulation of the Bmp‐Smad pathway 39 and also under the regulation of the classic mesendodermal marker Brachyury (or T), upregulated by Bmps from the extra‐embryonic regions 40.

## What is known on PGC specification in mammals other than rodents?

This complex regulatory network controlling pPGC and PGC formation in mouse is the only description of a molecular mechanism driving germ cell specification by induction in amniotes. Is the mechanism described in mouse employed by all mammals? This is a more‐than‐justified question if we consider that rodents stand out among mammals for the peculiar shape of the pre‐gastrulating embryo 10, 34. For example, *Bmp4* is expressed in the ring of extra‐embryonic ectoderm bordering with the epiblast, but this structure largely developed in rodents does not exist as such in non‐rodents 41. Even in the rodent guinea‐pig (*Cavia porcellus*), the extra‐embryonic ectoderm does not contact directly with the epiblast at the time of PGC induction 10. Moreover, murine PGCs nest in the growing mesodermal allantois in the proximal/posterior region of the embryo, a structure precociously developed in the mouse, but less so in non‐rodent mammals. In humans, the allantois is a mixed structure of endoderm and mesoderm developed to store waste products as in chicken, but unlike the mouse 42. Therefore, we need to take into account the unique morphological features of the mouse embryo, and consider the possibilities that the mechanism involved in PGC formation might have taken different molecular routes in non‐rodent mammals. The only way to shed light on this issue is to analyse and compare a diverse pool of mammalian embryos other than rodents.

It is still not known whether PGC specification in humans takes place within the first 14 days of development, the maximum number of days that scientists can keep human embryos from in vitro fertilization in culture. In vivo, the day‐14 human embryo is a flat disc that already has a well‐defined amniotic cavity and yolk sac cavity and is lined with extra‐embryonic mesoderm. The production of extra‐embryonic mesoderm starts around day 10 and the production of embryonic mesoderm (and the formation of the primitive streak) starts around day 15 43. It remains unclear when during this time window we can expect (pPGCs) and PGCs to be formed. As novel implantation models 44 and ‘gastrulation’ models using pluripotent stem cells are being developed 45, 46, these may prove to be useful assays to understand the molecular mechanisms that lead to PGC formation in humans. Moreover, directed PGC‐differentiation protocols applied to mouse and human pluripotent stem cells are starting to reveal species‐specific signals regulating specification of PGC‐like cells 47, 48, 49, even though in vivo data might be lacking.

On the basis of morphology, PGCs have been described in human embryos at early somite stage in the endoderm of the posterior end of the yolk sac, close to the allantois entrance, by Politzer and by Witschi (reviewed in Ref. 34). Decades later, ALPL activity in presumably PGCs was observed by several groups in human embryos with 5–8 somites (between 3 weeks of development or 5 weeks of gestation) in a similar location (reviewed in Ref. 34). The involvement of BMP4 as a PGC‐inducing signal in vivo in humans remains unclear, as well as the early lineage markers of pPGCs and PGCs. Using single cell analysis, human PGCs isolated at 4 weeks of development seem to express *PRDM14* and *TFAP2C*, whereas *PRDM1* and *IFITM3* are not expressed 50, suggesting that the critical molecular network in mouse and human early PGCs is divergent.

In the rabbit the early embryo is flat, a feature shared by the whole non‐rodent mammalian group, as far as we know 51. In the rabbit, PG‐2 (a germ cell epitope) and *Prdm‐1* expressing cells have been localised at early gastrulation stage in a region identified in the posterior upper layer (epiblast) and mesoderm 41, 52 (Fig. 2B). However, *Prdm‐1* presents a wider expression pattern during these developmental stages, with positive cells in the hypoblast all around the circumference of the embryo, adjacent to the site of *Bmp4* expression in the extra‐embryonic cells surrounding the embryo 53. However, from these ‘blimped’ pPGCs only the posterior ones seem to become PG‐2‐positive PGCs. The use of a wider array of molecular markers will be crucial to clarify the timing and location of appearance of pPGCs and transition to PGCs in the (pre)gastrulating rabbit embryo.

Other mammals have been analysed for the identification and distribution of germ cells, including pig 54, dog 55 and sheep 56. However, in these cases the analyses were limited to the identification of germ cells in the developing gonads or, like in the case of the pig, to the analysis of the distribution of pluripotency markers like *Pou5f1* and *Nanog* in pregastulating and gastrulating pig embryos 57. A recent report on the marmoset monkey looked at the later distribution of PGCs and speculated on alternative migration paths, but no data on the expression of the early PGCs markers as in mouse were described, due to the obvious paucity of specimens 58.

As far as marsupials (or methatheria) are concerned, the tammar wallaby *Macropus eugenii* has been the object of study to understand PGC migration from just after gastrulation until the arrival at the gonads 59, 60 and more recently the epigenetic reprogramming occurring in the PGC after colonization of the genital ridge 61. To date no information about molecular signals necessary for PGC specification and early localization is available for monotremes (or prototheria), the egg‐laying mammals.

Too few comparative studies have been conducted to allow speculation on a putative general molecular mechanism employed by mammals, or on modifications evolved to accommodate (or as a consequence of) the development of diverse topological organizations of the embryo. In the absence of comparative data, it is also impossible to infer any conclusion about the evolution of PGC specification from a urodele‐like amniote ancestor. Could the analysis of the other amniote branch, represented by the sauropsids (reptiles and birds), help us understand the evolutionary path of PGC specification?

## A historical perspective on avian PGCs: Is maternal specification the bird's way?

When the chicken egg is laid, the embryo, which already contains about 50,000 cells and is organised in a flat round single‐layered blastodisc (epiblast) can be cut in pie‐shaped slices, and each slice can generate an embryonic axis 62, 63, 64, 65, 66. This property lasts until the initiation of gastrulation, 12 hours after the egg has been laid, when a lower extra‐embryonic layer has grown below the epiblast and the embryonic axis, or primitive streak, is about to appear. Up to that time, the epiblast cells maintain a high degree of plasticity with regard to lineage specification, as in the mammalian embryo, suggesting the absence of any maternally produced determinants. Therefore, it would be reasonable to think that induction would be the modality adopted in PGC specification in avian embryos. Despite this regulative capacity, it was proposed that different ‘ooplasms’ (maternally derived substances present in the egg cell), localised in different regions of the mature oocyte, are inherited by different portions of the developing embryo: pPGCs would inherit the so‐called ‘δ ooplasm’, localised in the deep central area 67, 68. This was the first support of what became the current view on PGC specification in the avian embryo: maternally produced germ plasm determines PGCs. The pregastrulating embryo can be cut in slices, and each piece will regulate and form a new embryo; nonetheless, according to this view, PGCs are already fixed and escape this regulation. Are the data that generated this view exhaustive?

**Figure 3:**
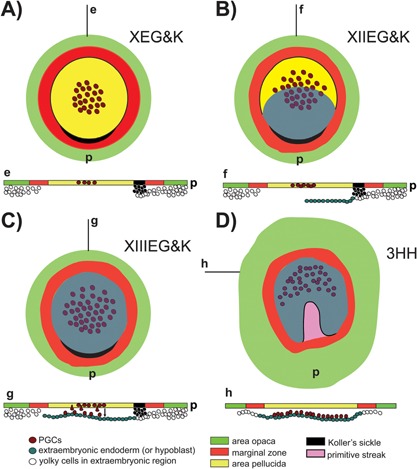
Dynamic localisation of pPGCs in the early chick embryo. Scheme summarising pPGC distribution in the early pre‐gastrulation (A, stage XEH&K) to mid‐gastrulating chick embryo (D, stage 3HH), and respective sections (e–h). All schemes show the ventral view of the embryos. **A** (e): Stage X embryo with pPGCs scattered in the central region of the epiblast layer. **B** (f): Stage XII embryo, with the growing hypoblast layer, and the pPGCs still in the epiblast. **C** (g): Stage XIII embryo, with a full‐grown hypoblast, which hosts pPGCs dropping from the epiblast. **D** (h): Stage 3HH (mid‐streak), pPGCs being displaced along with the hypoblast towards the germinal crescent, an anterior‐lateral horseshoe region evident at later stage (see Figure 2C). p, Posterior part of the embryo.

PGCs have been described by morphological criteria in 4‐day‐old chicken embryos since the end of the XIX century, but only Swift in 1914 described them in an anterior‐lateral ‘crescent’ position at the border between the embryonic (area pellucida) and extra‐embryonic (marginal zone) region in the late gastrulating chick embryo by means of morphological and cytological criteria 69 (Fig. 2C), a region that does not contribute to the embryo proper, hence suggesting an extra‐embryonic origin of PGCs. We had to wait another 60 years for the seminal work from Eyal‐Giladi and coworkers 70, 71, 72, 73, 74 to learn that PGCs have an embryonic origin, being localised exactly in the central disc of the single‐layered epiblast of the freshly laid egg (stage X EG&K) 71 (Fig. 3A). They also mapped the path that the PGCs take to reach the lower layer of the germinal crescent: from the epiblast where they can be found until the beginning of gastrulation, PGCs start ‘dropping’ onto the newly formed extra‐embryonic lower layer, the hypoblast, and, together with it, are translocated anteriorly to the ‘germinal crescent’ where they were localised using the periodic acid‐Schiff (PAS) colorimetric reaction (Fig. 3B and C). This process lasts until middle‐late gastrulation, when most of the PGCs have been removed from the epiblast layer (Figs 2C and 3D). Interestingly, the impossibility of observing PGCs in the early pre‐gastrulation stages, and their appearance after the body axes have been laid down has been interpreted as a sign of the absence of any germ plasm, and in support of induction as the modality for PGCs specification in chick 72. As germ plasm could not be identified in chick, in the first and only attempt to kill PGCs, Fargeix irradiated by X‐ray the whole epiblast of un‐incubated duck embryos; he then let them grow until the 10‐somite stage, counted the number of PGCs and registered a general decrease 75. However, the irradiation treatment, being quite an aggressive and non‐specific procedure, may have influenced cell death and development of the whole embryo and not only of the PGCs (the general state of the embryo was not described 75).

As mentioned, the early pre‐gastrulating chick embryo is highly regulative in the sense that once an embryo is cut in half, each half develops an embryo independently of the other half. This type of experiment has been utilised to verify the possible re‐induction of PGCs, but attempts by different groups have given opposite results. On the one hand, isolated anterior and posterior halves from duck 76, 77 and chick embryos 72 regulated the number of PGCs, and half embryo produced a number of PGCs equal to that of a normal intact embryo. However, in a later attempt of the same experiment using chick, no regulation was observed, and the total number of PGCs in anterior and posterior halves equalised the number of PGCs in control uncut embryos 74, 78. The question of PGC specification in avian still remained open.

An important advance in resolving this issue was provided by Tsunekawa and coworkers 79, who developed the first germline‐specific antibody against chicken Ddx4 (or chicken vasa homolog, CVH). They were in fact able to identify Ddx4‐positive cells in very early stages of embryonic development (before the egg is laid). Ddx4, an ATP‐dependant RNA helicase, localised to the cortex of growing oocytes, and then to a globular structure the authors identified as the mitochondrial cloud in mature oocytes. At two‐cell stage – and for the next few divisions – in chicken embryos, Ddx4 was observed in the proximity of the cleavage furrow and became localised to the ventral portion of the blastomeres from stage IV EG&K on. At oviposition, Ddx4‐positive cells were found scattered in the central area of the epiblast 79. The early localization of this germline marker prompted Tsunekawa and coworkers to support a maternal specification mode of PGC formation in chick in which Ddx4 is part of the germ plasm, as in zebrafish 80. This idea has been accepted and is nowadays rooted in the field.

Recently, the protein and RNA expression pattern of another germ plasm‐germline marker *Dazl*, encoding an RNA binding protein, has been reported in early chicken embryos 81. Accordingly, *Dazl* mRNA was found in the cortex of maturing chick oocytes and in the first cleavage furrow. However, by stage IV EG&K, *Dazl* mRNA distributed diffusely to the cytoplasm of the blastomeres. Therefore, the authors suggested that early *Dazl* is maternally inherited, but the later diffused distribution may result from embryonic transcription, as embryonic gene activation initiates at stage II–III EG&K 82, leaving open the possibility that a mechanism of induction from the nearby somatic cells could contribute to PGCs specification.

**Figure 4:**
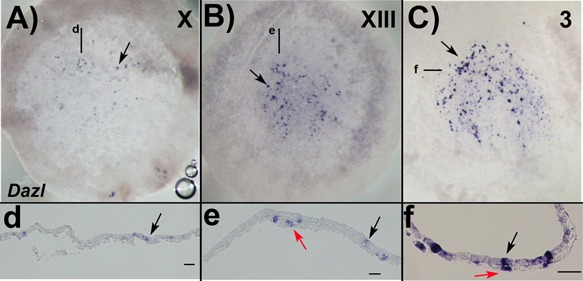
Expression pattern of *Dazl* during early chick embryo development. Stage X EG&K (**A**), XIII EG&K (**B**), and 3HH (**C**), showing pPGCs scattered distribution in the embryonic area pellucida (arrows). Sections show pPGCs exclusively in the upper layer at stage X (**d**), while at stage XIII (**e**) and 3 (**f**) they localise both in the upper (black arrows) and in the lower (hypoblast) layer (red arrows). Posterior is at the bottom in whole‐mount (A–C), and on the right in sections (d and e). Scale bars: 50 μm.

Is the distribution of PGC markers Dazl and Ddx4 in sauropsids sufficient to draw conclusions over the mode of PGC formation? From stage X EG&K to Hamburger and Hamilton stage 3(HH) chick embryos, *Dazl* RNA is scattered in the epiblast (Fig. 4A and B) in no apparent overlap with the reported DDX4 protein distribution (clearly concentrated at the boundary with the area opaca) 79, whereas colocalization of Dazl and Ddx4 was clearly the case at stage 7HH 81. Furthermore, at early streak stage in chicken, a considerable amount of *Dazl* expressing cells are still in the epiblast and not in the hypoblast, pointing towards the existence of two different populations (Fig. 4C). This heterogeneity in the expression of germline markers is also observed in early stage PGCs both in mouse 7, 83 and human 84, and may be a general characteristic of pPGCs and PGCs. Investigating the expression and localization of additional germ plasm markers, including DND1, TDRD, NANOS and PIWI 26, 85, 86, 87, would help clarify the presence of germ plasm in chicken.

## What do we know of reptiles other than birds?

PGCs have been localised via morphological and cytological landmarks in the extra‐embryonic lower layer (or hypoblast) of a few species from late gastrulation/early somite stages, in diverse locations along the anteroposterior axis. In the snake *Vipera aspis* 88, PGCs were found in the hypoblast, to be translocated to the (anterior) germinal crescent during gastrulation, as in birds. In other Squamata, such as the lizards *Mabuya megalura*, and *Anguis Fragilis* or the chameleon *Chamaeleo bitaeniatus*, the PGCs were identified in both the anterior and the posterior regions, surrounding the embryo (‘circumferential’) 88, 89, similar to what has been described in the *Sphenodon punctatus* 90. However, among reptiles, examples of PGC distribution posteriorly have also been reported in *Lacerta vivipara* jacquin 91 and turtles. In the latter, PGCs had been localised only in early somite stage turtle embryos, and consistently in the posterior and lateral hypoblast in different species (*Chrysemys marginata* 92, 93, *Caretta caretta* 94, 95, *Sternotherus odoratus* 96).

Recently, PGC localization in early stages of development of the turtle *Trachemys scripta* has been described with the help of molecular markers 97. *Dazl* and *Ddx4* mRNA are expressed both in a structure called the blastopore plate, located posterior to the blastopore supposedly containing prospective mesendoderm cells, and in the lower layer (definitive endoderm or hypoblast) 97, 98, 99. These expression patterns and the absence of localised RNA for the two germ cell markers in turtle mature oocytes suggests that germ plasm is indeed absent in turtles, and an inductive modality of PGC specification might be employed.

The data reviewed for birds and reptiles highlight a lack of certainty and the obvious need for deeper analyses, as it is nonetheless an accepted believe that that avians (maternal specification) and reptiles (induction) diverge regarding PGC formation.

## Could certain PGC features be associated with the mode of PGC formation?

PGC localization at gastrulation could perhaps provide an evolutionary framework in the understanding of diversities or affinities in the mode of PGC formation. From an embryological point of view, both chicken (avians) and viper snakes (reptiles) employ a novel strategy in which pPGCs migrate first anteriorly to the evolutionary novel structure germinal crescent, before entering the circulation en route to the gonads 8, 100, 101, and could have adopted the more recent maternal determination mode. However, in chicken the pPGCs drop from the epiblast center 70, 81, whereas in vipers pPGCs seem to delaminate from the posterior blastopore 88. In addition, several lizards, chameleons and skinks show a ‘circumferential’ distribution of pPGCs, and also seem to employ a ‘circumferential’ vascular route to reach the gonads 8, 89, 101, 102.

By contrast, in turtles, the only other non‐mammalian amniote for which a reasonable molecular data set exists, the posterior distribution of PGCs in gastrulating embryos resembles the posterior localization of mouse PGCs. Moreover, the blastoporal plate region in turtles also expresses *Brachyury* (as does the mouse primitive streak) 98 and turtle PGCs migrate interstitially to the gonads 97. In that regard, the embryology of turtles resembles that of mammals and urodele amphibians, and reflects the urodele‐like amniote ancestor, suggesting that the mode of PGC formation could also be conserved (induction). However, the scanty and diverse data available for other reptiles indicates a high degree of variability, in which PGCs are distributed anteriorly, posteriorly or circumferentially at gastrulation, depending on the species 97. Comparative embryology among sauropsids does not help reduce the existing diversity to the binary mode of PGC formation.

Furthermore, if we look at mammals, while in mouse ‘blimped’ pPGCs and PGCs segregate posteriorly, in the rabbit, ‘blimped’ pPGCs localise anteriorly as well, in a fashion not dissimilar to that seen in birds. This criterion is therefore unreliable for a comparative analysis that could give insight into the evolution of PGC specification and help to predict the mode of PGC formation.

While in the mouse transplantation/deletion experiments have been conducted to show that prospective non‐PGCs can be reprogrammed to become PGCs if grafted in the proper region at the proper time 7, 9, in sauropsids (reptiles and birds), particularly in the chick, such experiments have not been attempted: the reason is the lack of suitable molecular markers and/or transgenic strains. Although we agree that the early localised expression of Ddx4 and Dazl, which are both markers of germ plasm in teleosts (*D. rerio*), anuran amphibians (*X. leavis*), insects (*D. melanogaster)* and nematodes (*C. elegans*), points towards maternal specification of avian PGCs, it is conceivable that such maternally inherited material may not be a proper determinant, and induction may still occur at later developmental stages. In this respect, it is notable that the distribution of the pluripotency markers *Nanog* and *PouV* (a *Pou2*‐variant in birds; *Pou5f1* in mouse 103) in chick is comparable to that observed in mouse. Both RNAs are ubiquitously expressed in the early epiblast and at late gastrulation stage they are expressed in the PGCs only 104, 105.

## Mode of formation: PGCs or pPGCs?

It is becoming clear that the concept of pPGCs and PGCs needs some re‐structuring and clarification. The term ‘pPGCs’ was introduced by Nieuwkoop and Sutasurya 8 and defined as forerunners of the PGCs, but it has remained unused until recently with the discovery of ‘blimped’ Blimp1‐expressing pPGCs in mouse 1. pPGCs are those precursors that by mitosis originate PGCs, but can adopt different fates if placed (or transplanted) in a different niche in the embryo. And in that sense, pPGCs can be considered latent multipotent progenitors; whereas PGCs retain their identity even if transplanted 7, 9 and certainly during their migratory phase to the gonads. Moreover, mouse ‘blimped’ pPGCs do not express most of the early PGC markers, including Dppa3 or show the ‘golden standard’ Alpl activity. Furthermore, if BMP4 is involved in ‘blimping’ pPGCs at E6.25 (when it is expressed), what are then the signals necessary to induce progression from (Alpl‐negative) pPGC to (Alpl‐positive) PGCs at E7.5?

To our surprise, *Xenopus laevis* pPGCs from tadpole stage, upon grafting in an early embryo will generate lineages of the three embryonic layers, not only germ cells 27, 106. This indicates that those pPGCs that are supposedly maternally specified are in fact not bona fide fate‐restricted PGCs. More recently, elegant transplantation experiments in *Xenopus* grafting (GFP) transgenic germ plasm to ectopic locations demonstrated that those ectopic pPGCs (carrying GFP‐positive germ plasm) could only develop to PGCs (and become mature gametes) when transplanted back to the original environment 27. The authors of that paper concluded that ectopic pPGCs would still require adequate signals from the endogenous environment to develop normally, suggestive of an inductive step from the surrounding tissue. It therefore remains unclear whether an induction mechanism (albeit to trigger migration or correct development) is also needed in the frog.

If this is our current scenario, the clear‐cut difference between maternally specification and induction as modalities of PGC formation becomes blurred, as even animals with maternal specification, may need additional inductive mechanisms for the lineage restriction step from pPGCs to PGCs. The discussion on PGC formation de facto becomes a discussion on pPGC formation and the mechanisms driving pPGCs emergence.

The ongoing development of technologies to characterise transcriptome and methylome profiles at single cell level may help generate a comprehensive framework whereby the signature of pPGCs will be distinguished from that of PGCs. Moreover, this novel technology will make it possible to benchmark and directly compare single‐cell transcriptional data of orthologue genes from germ cells from different species and isolated at different time points. Starting with species like the mouse, zebrafish, *X. leavis*, *C. elegans* and *Drosophila* may help create predictive transcriptional signatures to evaluate the mode of PGC formation of other species.

Alternatively, what determines that a PGC is different from a (multipotent) pPGC may not be revealed by the transcriptome, but rather reflected in the levels (and/or the pattern) of DNA methylation. Both high‐throughput genome‐wide single‐cell transcriptomics and methylomics are fast evolving techniques 50, 84 that will allow a detailed molecular analysis and comparison between species that will lead to a clarification on the modality of pPGC and PGC formation.

## Conclusion and outlook

The revision of data available on PGC specification in amniotes sheds doubts on the current tendency to divide embryos in two clear‐cut categories, the ones adopting maternal specification (predetermination) versus the ones employing induction (epigenesis), as previously suggested from studies focussing on non‐amniotes 3, 107, 108. The only amniote species where the molecular mechanism of induction has been dissected is the mouse. No demonstration of either mechanism has been provided so far in sauropsids, neither in birds nor in reptiles.

Does this duality reflect all of the possible cell behaviours? In an alternative scenario to the binary predetermination versus induction choice, we propose that a species‐specific continuum of multiple steps could be involved in germ cell bias, with PGCs and (multipotent) pPGCs coexisting early, and pPGCs requiring a further induction step to reach the PGC status. Maternal determinants, when present, would intervene in some unknown aspect of (multipotent) pPGC specification/retention (including inhibition of gene expression by transcriptional and translational repression).

In the animal kingdom, vertebrates and ecdysozoans (such as *C. elegans* and *Drosophila*) are thought to adopt an early soma‐germ line segregation, while other taxa maintain a multipotent population of cells which will give rise to germ cells in the adults 107, 108. We propose that even when the early segregation occurs, multipotent progenitors (presumably pPGCs) are established in the embryo, regardless of the defined modality of PGC formation adopted by the species. These multipotent precursors can give rise to the germ line either during embryonic development or adult life 3, 107, 108. The question remains as to when and by which mechanism those pPGCs are established (retention of maternal transcripts, germ plasm, induction etc). In this regard, we speculate that the establishment of (multipotent) pPGCs may be a common theme in all animals, with a species‐specific twist on how and when pPGCs are established.

The authors have declared no conflicts of interest.
